# Behavior of earth-rock cofferdam built on deep overburden foundation considering seepage effect

**DOI:** 10.1371/journal.pone.0361805

**Published:** 2026-10-30

**Authors:** Linsong Zhu, Shuangquan Li, Xuewu Sun, Yuechen Wei

**Affiliations:** CCCC Hehai Engineering Corporation Limited, Taixing, China; Northeastern University, CHINA

## Abstract

An increasing number of water conservancy and hydropower projects are being constructed on deep overburden foundations. Deep overburden foundations have an important influence on the mechanical behavior of earth-rock cofferdams built on them. A three-dimensional coupled seepage–stress finite element model was established to compare the two schemes under different cutoff-wall penetration depths and rapid-drawdown conditions. This paper presents a comparative analysis of the seepage and mechanical behavior of earth-rock cofferdams built on deep overburden foundations with two seepage-control schemes, namely, a composite geomembrane core-wall scheme and a composite geomembrane inclined-wall scheme. The seepage-control performance, stress and deformation characteristics, and slope stability of the cofferdams under the two seepage-control schemes are discussed. In addition, the behavior of the cutoff wall in the deep overburden under different conditions is compared. The results show that when the cutoff wall completely penetrates the overburden, it can effectively reduce the total head and lower the phreatic line in the cofferdam. For all cutoff wall depths, the seepage flow of the core wall scheme is lower than that of the inclined wall scheme. The stress distribution patterns in the cofferdam and cutoff wall are essentially the same for the core wall and inclined wall schemes. However, the tensile stress is slightly higher in the core wall scheme. During water level drawdown, the safety factor of the cofferdam is generally higher for the inclined wall scheme than for the core wall scheme.

## 1 Introduction

More and more earth-rock cofferdams must be built on deep overburden foundations as a result of the growing number of water resources and hydropower projects. These deep overburden layers are widely distributed in the upper and middle reaches of the Jinsha River, the upper reaches of the Nujiang River, the main stream and tributaries of the Dadu River, as well as some rivers in Tibet and Xinjiang. Deep overburden layers are characterized by non-uniform mechanical and physical properties, a loose structure, and discontinuous gradation. Seepage control, structural safety, and stability are among the main challenges faced by earth-rock cofferdams constructed on such foundations [1]. To ensure the safety of these cofferdams, it is essential to investigate their seepage behavior, stress–deformation characteristics, stability, and seepage-control schemes under deep overburden foundation conditions.

Numerous studies have investigated seepage characteristics in cofferdams. Deng et al. [2] used the finite element method to investigate the variation patterns of the seepage and stress fields during foundation pit dewatering. They focused on the overall deformation of the cofferdam, as well as the shear failure and instability characteristics of its slopes. Hu et al. [3] carried out numerical analyses to study the coupling effect between the cushion layer and the downstream concrete slab, and derived a limit equilibrium equation for seepage failure in the cushion layer. This study provides a theoretical basis for the structural design of downstream concrete slabs in earth-rock cofferdams. Wang et al. [4] investigated the seepage characteristics of dam foundations on deep overburden considering the spatial variability of permeability and evaluated the effects of different cutoff-wall depths on hydraulic gradients and seepage flow. Xiong et al. [5] compared different seepage-control schemes for an earth-rock cofferdam using finite element seepage analysis and demonstrated that the arrangement of anti-seepage structures significantly affects seepage-control performance. Overall, existing studies have improved the understanding of seepage-induced deformation, local seepage failure, and structural stability in earth-rock cofferdams. However, most studies have focused on specific seepage responses or individual structural components, while systematic comparisons of different anti-seepage schemes for cofferdams founded on deep overburden layers remain limited. Therefore, further investigation is needed to evaluate the overall effectiveness of different seepage-control schemes under deep-overburden foundation conditions.

Other studies have focused on the stress–deformation and stability characteristics of cofferdams. Liu et al. [6] used the strength reduction method and FLAC2D to evaluate the safety of cofferdam cross-sections under two conditions. Their results showed that the addition of counterweight platforms can significantly improve cofferdam safety. Ayiheng et al. [7] used ABAQUS to study the stability of earth-rock cofferdams built on deep overburden foundations under seepage conditions. Their study showed that the installation of vibro-replacement stone columns before excavation can improve foundation pit drainage, making this method suitable for earth-rock cofferdams built on deep overburden foundations. Wang et al. [8] used Bayesian methods to investigate the uncertainty in the overtopping behavior of earth-rock cofferdams and to predict the probability of failure through parameter back-analysis. Xue et al. [9] carried out model tests on cofferdams to examine their deformation under different water levels, and established a three-dimensional numerical model to evaluate stability. Their results showed that the numerical simulations agreed well with the measured data. Yu et al. [10] investigated the stress–deformation behavior of concrete anti-seepage structures in earth-rock dams on overburden foundations and showed that the spatial variability of foundation parameters can significantly affect structural stress and deformation. Halim et al. [11] evaluated the slope stability of a cofferdam using finite element analysis under different water-level conditions, including rapid drawdown, and assessed the corresponding changes in the factor of safety. Overall, existing studies have advanced the understanding of cofferdam stability, deformation, and failure risk under different conditions. However, most studies have focused on overall stability or specific reinforcement measures, while the mechanical responses of different seepage-control schemes under deep-overburden foundation conditions remain insufficiently compared. Therefore, a systematic evaluation of their stress–deformation behavior and stability is still needed.

Based on a three-dimensional coupled seepage–stress finite element model, this study compares the composite geomembrane core-wall and inclined-wall schemes for an earth-rock cofferdam founded on deep overburden. The analyses consider different cutoff-wall penetration depths and rapid-drawdown conditions, and evaluate seepage-control performance, stress–deformation responses of the cofferdam and cutoff wall, and slope stability.

## 2 Calculation method

### 2.1 Coupled seepage-stress field analysis

Loading-induced compression of the cofferdam and foundation alters the pore volume and, in turn, the hydraulic conductivity of the granular materials. The coupled analysis therefore updates permeability as deformation develops. Let *n*_*0*_ denote the initial porosity and let the volumetric strain *εv = ΔV/V* arise from the change in pore volume, where *V* is the total volume of the soil element and *ΔV* is the corresponding pore-volume increment. The permeability-porosity relationship is represented by a modified Kozeny-Carman expression [12]:


$$\begin{array}{c} \left\{ \begin{array}{cc} & n_{p}=n_{\text{0}}+\varepsilon_{v}\\ & k_{\text{0}}=k^{\prime }{\left\{\frac{\left(n_{\text{0}}+e_{v})(\text{1}-n_{\text{0}}\right)}{n_{\text{0}}(\text{1}-n_{\text{0}}-e_{v})}\right\}}^{\text{3}} \end{array}\right. \end{array}$$ (1)

where *n*_*0*_ is the initial porosity, *n*_*p*_ is the porosity after deformation, *k*_*0*_ and *k’* are the permeability coefficients associated with *n*_*0*_ and *n*_*p*_, respectively, and *ε*_*v*_ is the volumetric strain of the soil element.

The interaction between the solid skeleton and pore water is described within Biot’s consolidation framework. In this formulation, saturated soil is idealized as a porous load-bearing skeleton whose pores are filled with water; the effective-stress principle links the mechanical field to pore pressure, while mass conservation links deformation to fluid flow [13].

Under the small-strain and fully saturated conditions adopted in this study, tensile stress is taken as positive and compressive stress as negative throughout this study. Under this sign convention, the total stress is decomposed into the effective stress carried by the soil skeleton and the pore-water pressure, and the effective-stress relationship can be written as:


$$\begin{array}{c} \sigma =\sigma^{\prime }-\alpha P_{w}I \end{array}$$ (2)

where σ and $\sigma^{\prime }$ are the total and effective stress tensors, respectively, α is Biot’s coefficient, $P_{w}$ is the pore-water pressure, and I is the identity tensor.

Under this sign convention, the force-equilibrium equations for an infinitesimal soil element can be written as follows [14]:


$$\begin{array}{c} \left\{ \begin{array}{cc} & \frac{\partial \sigma_{x}^{\prime }}{\partial x}+\frac{\partial \tau_{xy}}{\partial y}+\frac{\partial \tau_{xz}}{\partial z}-\alpha \frac{\partial P_{w}}{\partial x}=\text{0}\\ & \frac{\partial \tau_{xy}}{\partial x}+\frac{\partial \sigma_{y}^{\prime }}{\partial y}+\frac{\partial \tau_{yz}}{\partial z}-\alpha \frac{\partial P_{w}}{\partial y}=\text{0}\\ & \frac{\partial \tau_{xz}}{\partial x}+\frac{\partial \tau_{yz}}{\partial y}+\frac{\partial \sigma_{z}^{\prime }}{\partial z}-\alpha \frac{\partial P_{w}}{\partial z}-\rho_{sat}g=\text{0} \end{array}\right. \end{array}$$ (3)

where $\rho_{sat}$ is the density of the saturated soil and *Pw* denotes pore-water pressure; $\partial \sigma_{x}^{\prime }$, $\sigma_{y}^{\prime }$, and $\partial \sigma_{z}^{\prime }$ are the effective normal stresses; $\tau_{xy}$, $\tau_{yz}$, and $\tau_{xz}$ are the shear stresses;

A second governing relation is obtained from fluid continuity. For a saturated element, the net volume of water entering or leaving the pores must be compatible with the volumetric change of the soil skeleton. According to Darcy’s law, the seepage velocity in each principal direction is proportional to the corresponding hydraulic gradient. The continuity condition requires the divergence of the seepage flux to be balanced by the rate of volumetric deformation of the soil skeleton. Combining this requirement with Darcy’s law gives the pore-pressure form of the flow equation:


$$\begin{array}{c} \frac{\partial \varepsilon_{v}}{\partial t}=k_{x}\frac{\partial^{\text{2}}P_{w}}{\partial x^{\text{2}}}+k_{y}\frac{\partial^{\text{2}}P_{w}}{\partial y^{\text{2}}}+k_{z}\frac{\partial^{\text{2}}P_{w}}{\partial z^{\text{2}}} \end{array}$$ (4)

where *k*_*x*_, *k*_*y*_ and *k*_*z*_ are the permeability coefficients in the *x*, *y* and *z* directions, respectively.

The mechanical equilibrium equation and the seepage continuity equation are solved as a coupled system. In physical terms, the external load is balanced by the internal forces generated by skeleton deformation together with the seepage-force contribution. The resulting differential equation for the coupled seepage and stress fields can be expressed as:


$$\begin{array}{c} \left\{ \begin{array}{cc} & -[\partial {]}^{T}[D][\partial ]\{w\}+[\partial {]}^{T}\{M\}P_{w}=f_{s}\\ & \frac{\partial }{\partial t}\{M\}[\partial ]\{w\}-k_{\text{0}}\nabla^{\text{2}}P_{w}=\text{0} \end{array}\right. \end{array}$$ (5)

where [*D*] is the constitutive matrix; {*w*} is the displacement-component vector; {*M*} = [1 1 1 0 0 0] *T; f*_*s*_ represents the seepage force; [∂] is the differential operator matrix and $\nabla^{\text{2}}$ is the Laplace operator.

In the coupled solution, displacement and pore-water pressure are treated as the primary field variables. Changes in pore pressure modify the effective stress and therefore the deformation of the soil skeleton, whereas the resulting volumetric strain updates the porosity and permeability through Eq. (1). The updated hydraulic properties are then used in the subsequent seepage calculation, thereby establishing the two-way coupling between the seepage and stress fields.

### 2.2 Contact surface model

Because the foundation overburden is loose and lithologically discontinuous, relative movement can develop between the soil and the concrete cutoff wall. The wall is subjected to gravity, lateral earth pressure, water pressure, and interface friction, as illustrated in Fig 1. A zero-thickness frictional contact formulation was therefore introduced to reproduce separation, contact, and sliding along the soil-wall boundary without making the response dependent on a physical gap in the mesh. The contact relationship and normal contact treatment adopted in the model are illustrated in Fig 2.

**Fig 1:**
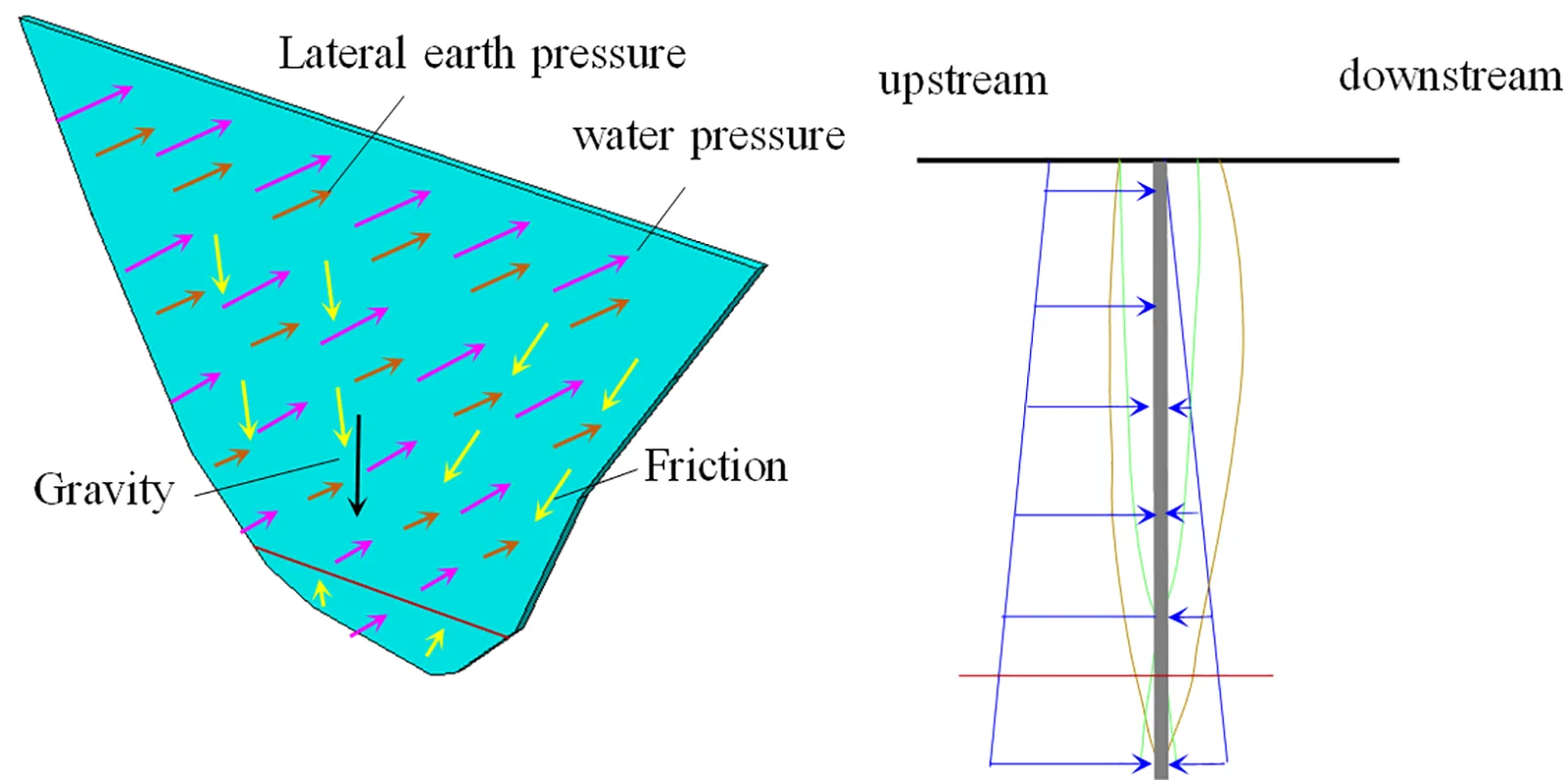
Mechanical characteristics of cutoff wall.

**Fig 2:**
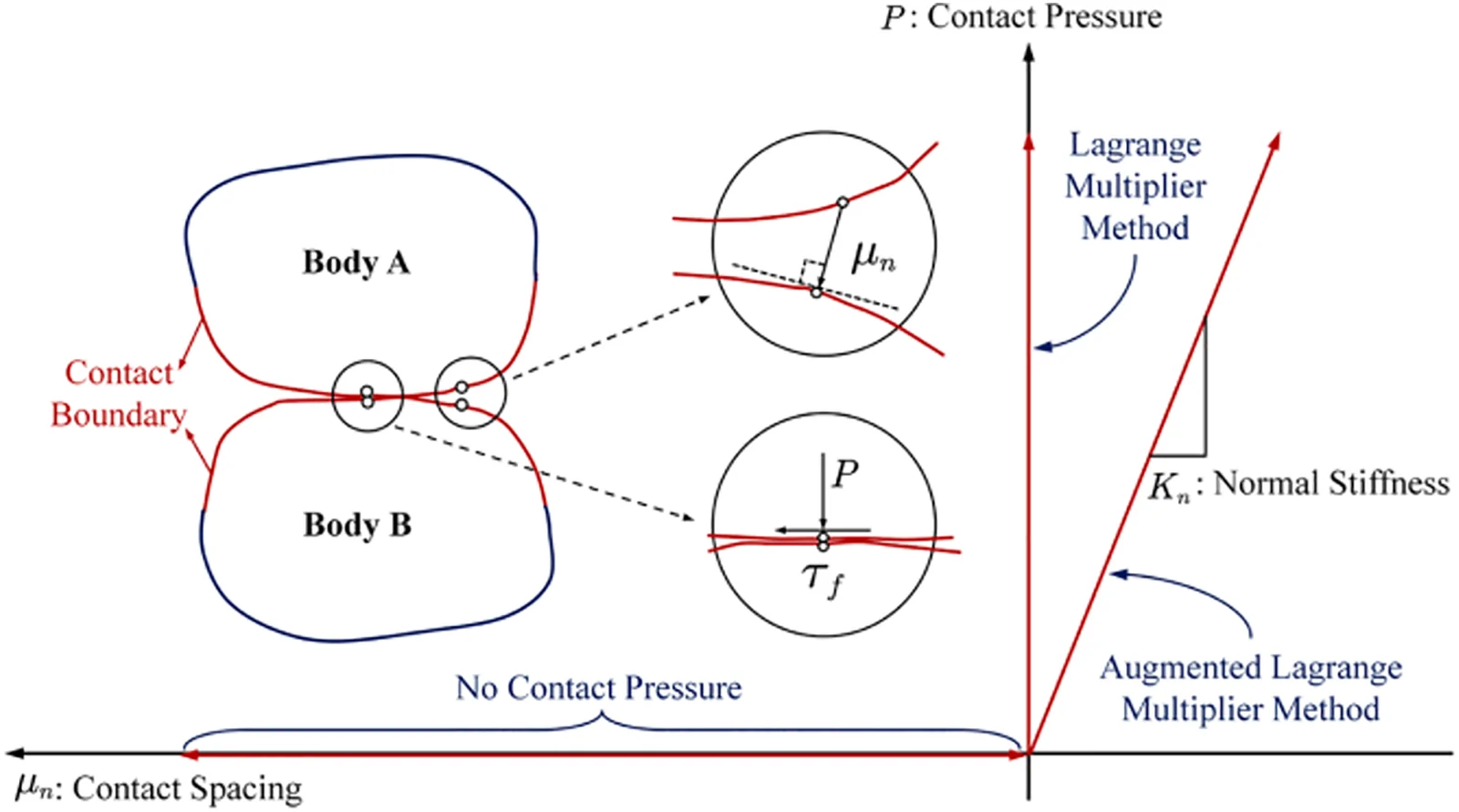
Contact surface between two objects.

The interface law contains independent normal and tangential components. In the normal direction, the interface is allowed to separate but excessive penetration is prevented once contact is established. In the tangential direction, the interface remains in a sticking state when the shear traction is below the limiting shear strength and transitions to sliding once the Coulomb friction limit is reached. This formulation therefore reproduces the opening–closure and stick–slip behavior of the soil–wall interface. The normal contact pressure P is evaluated using the following relation [15]:


$$\begin{array}{c} P=\left\{ \begin{array}{cc} \text{0} & ,\mu_{n}>\text{0}\\ K_{n}\mu_{n}+\lambda_{i+\text{1}} & ,\mu_{n}\leq \text{0} \end{array}\right. \end{array}$$ (6)

$$\begin{array}{c} \lambda_{i+\text{1}}=\left\{ \begin{array}{cc} \lambda_{i}+K_{n}\mu_{n} & ,\left|\mu_{n}\right|\leq \varepsilon \\ \lambda_{i} & ,\left|\mu_{n}\right|>\varepsilon \end{array}\right. \end{array}$$ (7)

where *μ*_*n*_ is the normal gap at the interface, *K*_*n*_ is the normal contact stiffness, *ε*_*0*_ is the allowable penetration tolerance, and *λ*_*i*_ is the Lagrange multiplier.

Tangential resistance along the interface is governed by a Coulomb-type friction law:


$$\begin{array}{c} \tau =f\sigma +c,|\tau |<\tau_{lim} \end{array}$$ (8)

where *τ* is the equivalent shear stress, *f* is the friction coefficient, *σ* denotes the magnitude of the compressive contact stress and is taken as a positive scalar in the Coulomb friction law, *c* is the interface cohesion, and *τ*_*lim*_ is the limiting shear strength. Accordingly, the limiting shear strength is controlled by the compressive contact stress, friction coefficient, and interface cohesion. When the interface shear stress is lower than $\tau_{lim}$, the contact remains in the sticking state; once τ reaches $\tau_{lim}$, relative sliding occurs along the interface.

The adopted interface formulation was checked against direct-shear data from the Zipingpu and Shuibuya projects [16]. For the Zipingpu simulations reported by Zhang and Zhang [17], the friction coefficient was 0.30 and the penetration tolerance was 0.5 mm; the corresponding Shuibuya simulations of Chen et al. [18] used the same friction coefficient and a 1.0 mm tolerance. As shown in Fig 3, the calculated shear stress-displacement curves reproduce the principal trends and stress levels measured in the tests. This agreement indicates that the zero-thickness contact model is suitable for representing the dominant mechanical behavior of the soil-structure interface.

**Fig 3:**
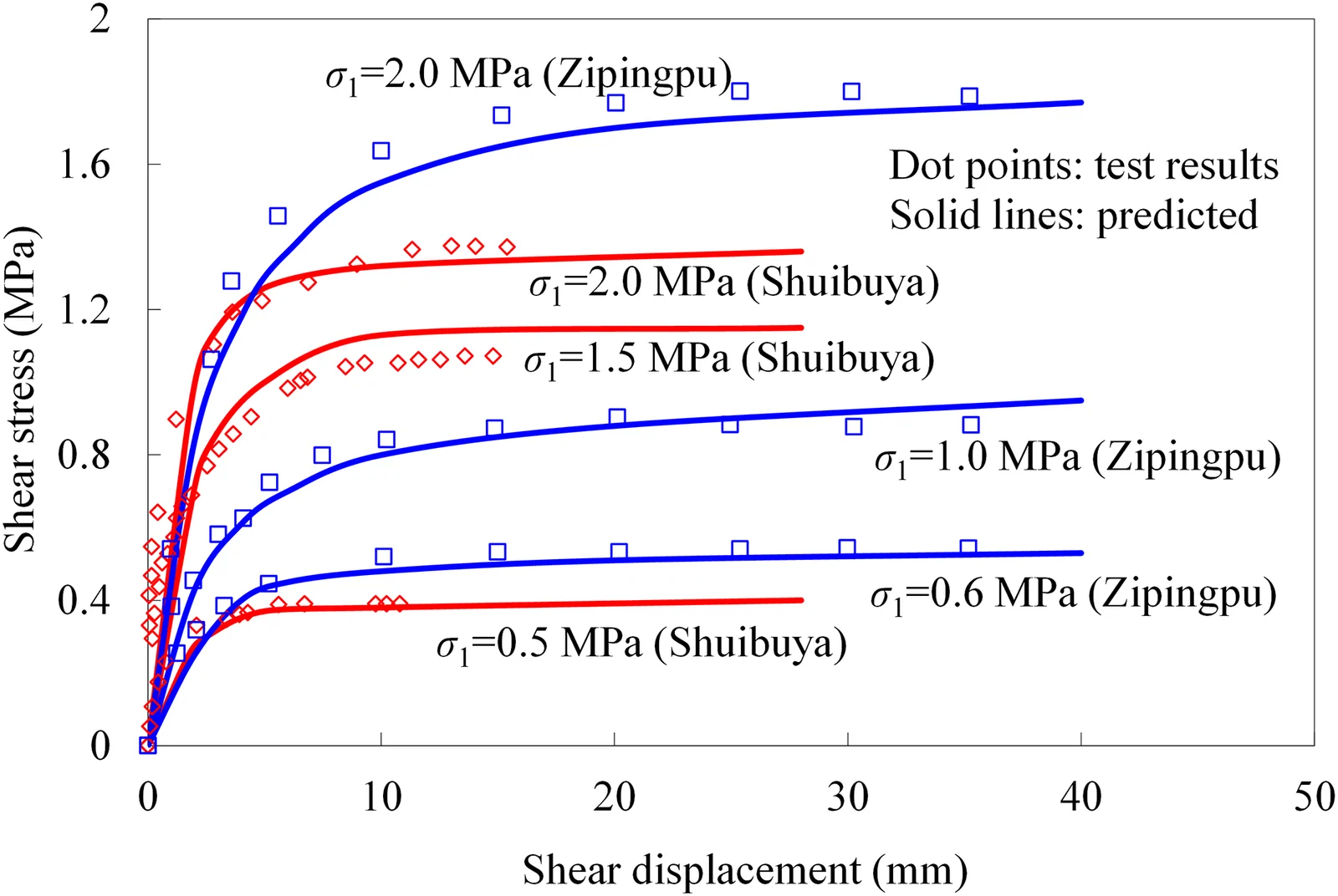
Comparison between simulation results of shear test and test results.

## 3 Project overview

The case study is the proposed upstream earth-rock cofferdam for the Zhongyu Hydropower Station on the Yigong Zangbo River. At the dam site, the controlled catchment area is 8,744 km^2^ and the mean annual discharge is 290 m^3^/s. The station has a planned installed capacity of 1,020 MW and is classified as a Class I large (1) project. The river is closed by the cofferdam arrangement shown in Fig 4. Under the 20-year construction-flood criterion, the upstream design water level is 3,057.90 m. Allowance for a possible glacial-lake outburst flood raises the cofferdam crest to elevation 3,075.00 m. The crest is 15 m wide and the maximum cofferdam height is approximately 55.0 m. The concrete cutoff wall is 1.2 m thick and reaches a maximum depth of 71.70 m. The upstream and downstream slopes are 1:1.75 and 1:1.5, respectively.

**Fig 4:**
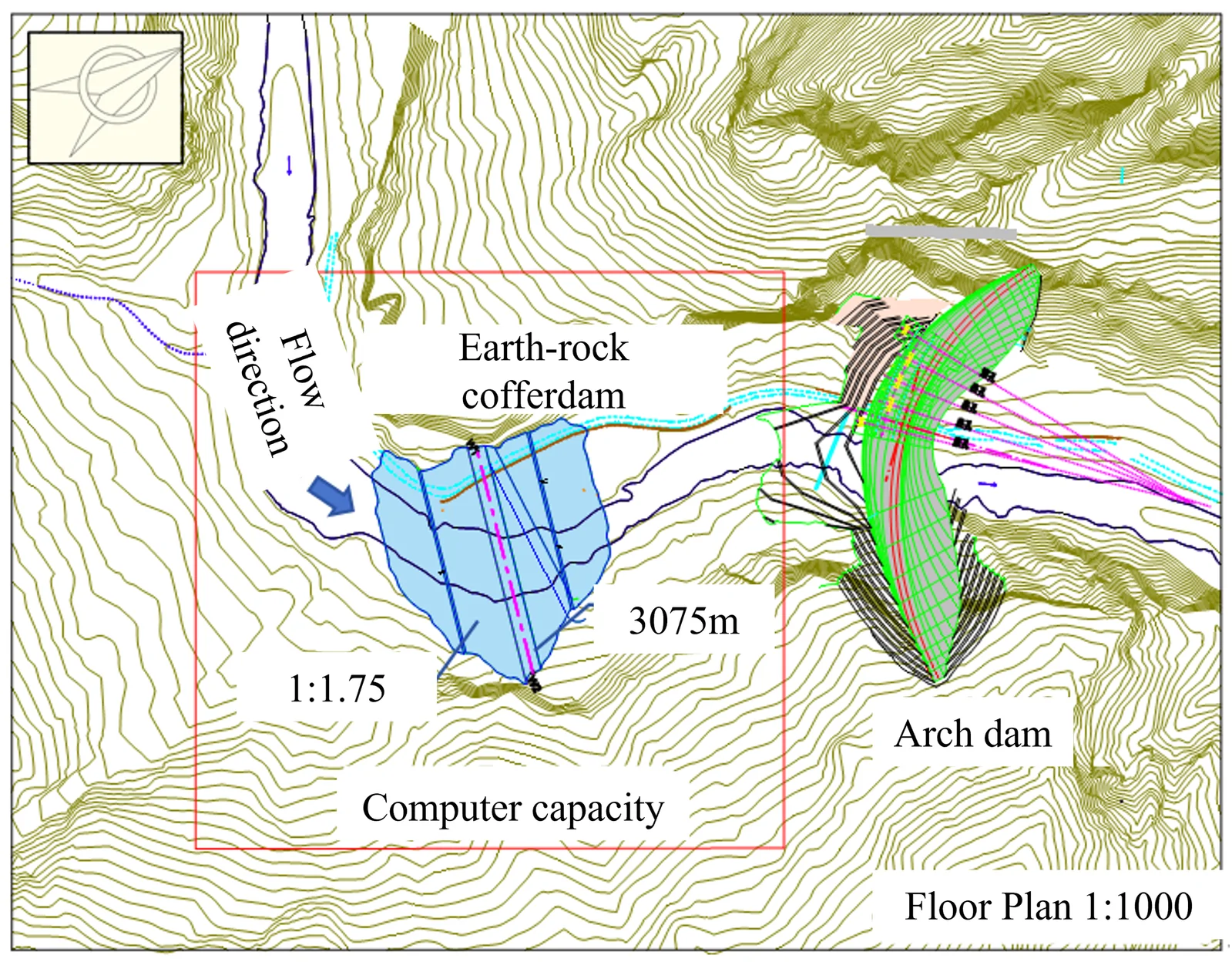
Layout of the project area.

The foundation profile at the upstream cofferdam is presented in Fig 5. During the normal-flow season, the channel is about 50 m wide. The riverbed is covered by a 45–60 m thick deposit dominated by sandy gravel containing cobbles and boulders, beneath which lies monzonitic granite. Strongly weathered rock is absent from the channel bed, while the moderately weathered zone is generally 5–12 m thick. Bedrock crops out on the left bank. On the right bank, a 25–35 m thick cover of blocky gravel and sandy cobble overlies the rock, and the colluvial material contains large boulders and crushed rock in a loose fabric. The horizontal extent of moderately weathered rock on the two abutments is approximately 15–30 m. These conditions indicate a strongly nonuniform foundation in which both seepage paths and soil-wall interaction require explicit consideration.

**Fig 5:**
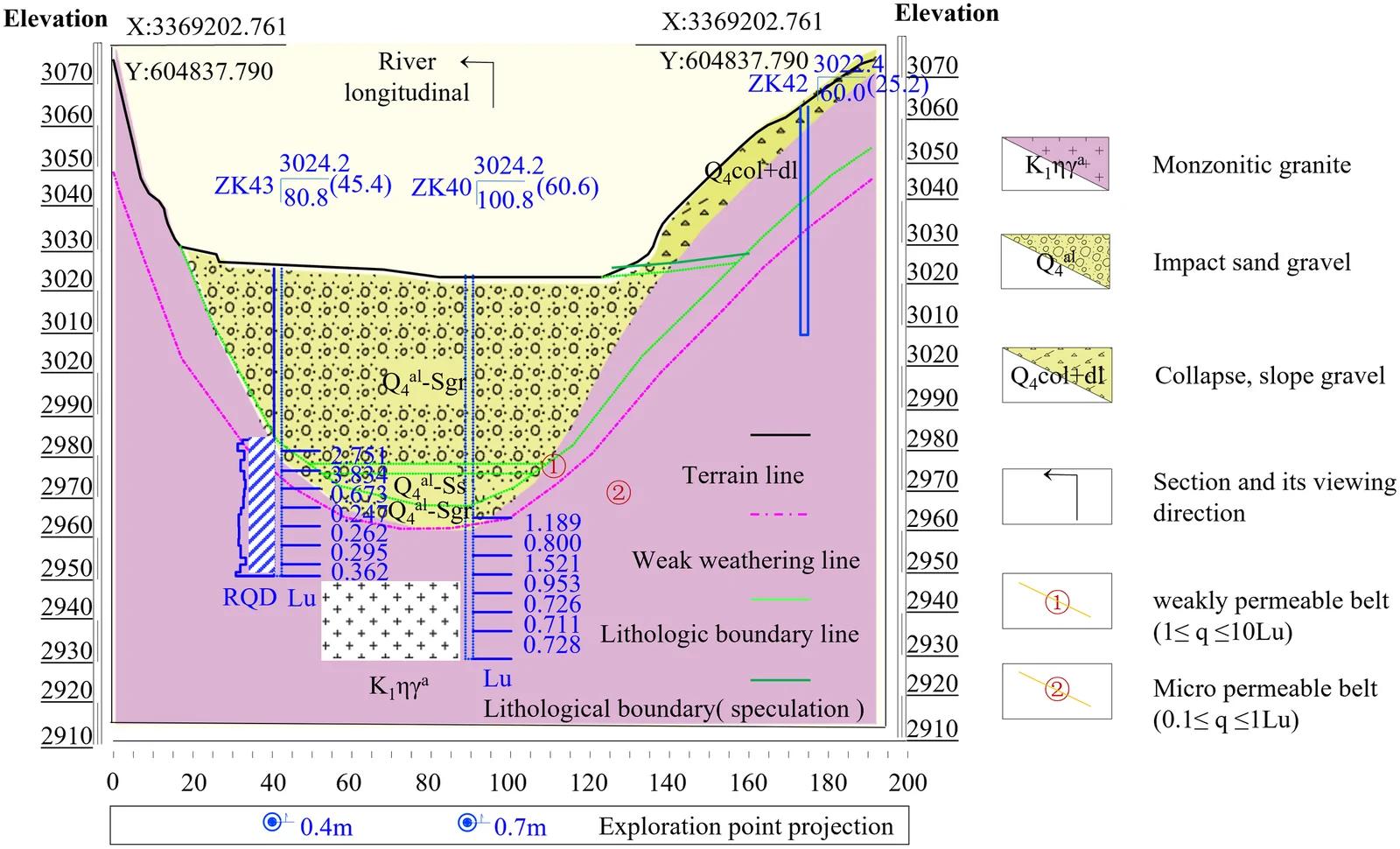
Geological conditions of upstream cofferdam.

Based on the engineering design requirements and the deep-overburden foundation conditions of the project, two alternative seepage-control schemes, namely a composite geomembrane core-wall scheme and a composite geomembrane inclined-wall scheme, were proposed during the design stage for comparison. Their representative sections are shown in Fig 6. In the core-wall arrangement, the cofferdam is zoned with stone-slag mixture, gravel, dumped clay, an interception dike, and a central composite geomembrane connected to the concrete cutoff wall. The inclined-wall arrangement uses the same principal fill zones, but relocates the geomembrane to the upstream face and shifts the concrete cutoff wall farther upstream. Thus, the principal distinction between the schemes is not the type of impermeable material, but the position and load-transfer path of the combined geomembrane-cutoff-wall system.

**Fig 6:**
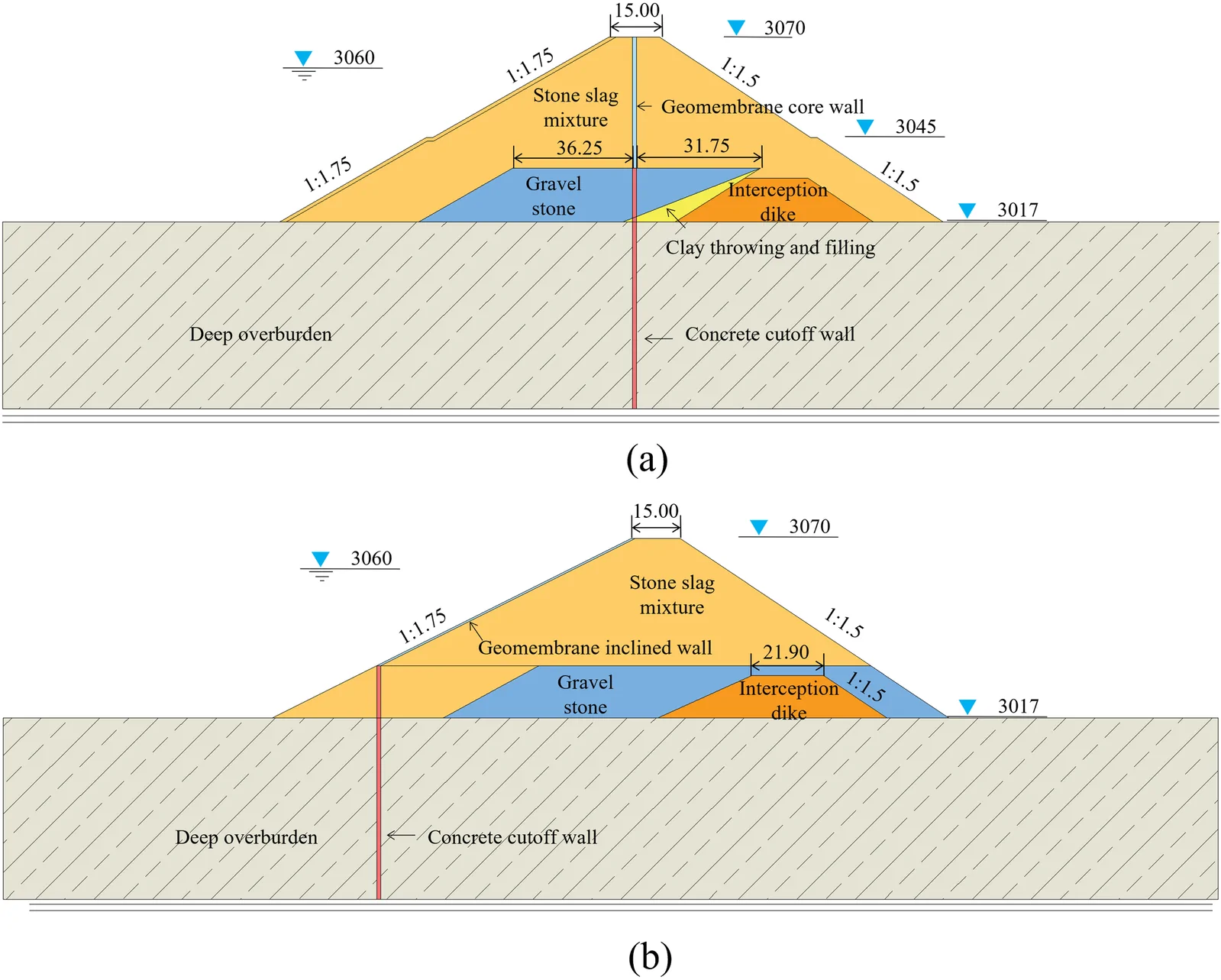
Typical cross section of earth-rock cofferdam (a) Core-wall scheme; (b) Inclined-wall scheme.

## 4 Calculation model and calculation scheme

### 4.1 Finite element model

A project-scale three-dimensional finite-element model was constructed for each seepage-control scheme (Fig 7). In every horizontal and vertical direction, the foundation domain extends approximately 1.5 times the cofferdam height beyond the structure. The base is fully fixed (U1 = U2 = U3 = 0); displacement normal to the upstream and downstream boundaries is restrained by U1 = 0, and the two lateral boundaries are assigned U2 = 0. The models explicitly include the overburden, weathered rock, fresh bedrock, and all cofferdam material zones. The core-wall mesh contains 136,575 nodes and 752,519 elements, while the inclined-wall mesh contains 136,762 nodes and 752,789 elements. Constant-head conditions are imposed on the upstream and downstream water boundaries and river channels. The model base and outer sides are treated as impervious, with potential seepage boundaries assigned elsewhere. This extent was selected to minimize the influence of artificial boundaries on the calculated seepage and stress fields while maintaining reasonable computational efficiency, following commonly adopted practices in similar finite element analyses.

**Fig 7:**
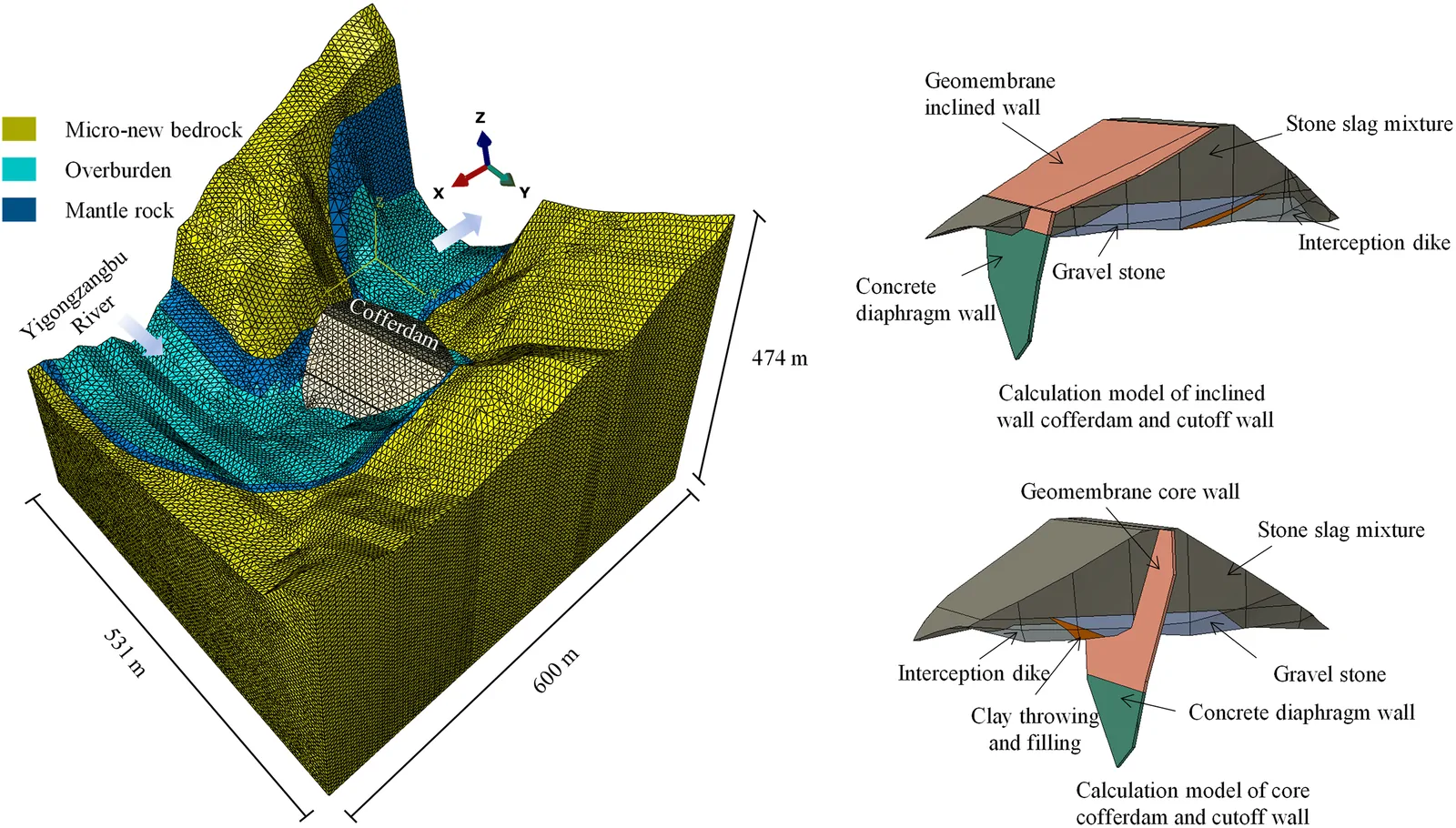
Finite element mesh and calculation model of two schemes.

### 4.2 Calculation parameter

The Duncan-Chang hyperbolic model is used for the cofferdam fills and overburden gravel, allowing tangent stiffness to vary with stress level during loading and unloading. The geomembrane, bedrock, and concrete cutoff wall are treated as linear-elastic materials. The adopted nonlinear and linear-elastic parameters are listed in Tables 1 and 2.

**Table 1:** Nonlinear material parameters.

Dam materials	*ρ*(kg/m3)	*ρ*_*sat*_	*K*	*n*	*R*_*f*_	*c*(kPa)	*φ*(°)	*K*_*b*_	*m*	*K*_*ur*_	*k (m/s)*	*J*
Interception dike	2080	2200	570	0.32	0.78	0	49.7	280	0.23	1140	3.0 × 10^−3^	0.2
Grave stone	2060	/	560	0.42	0.87	0	49.2	310	0.24	1120	1.0 × 10^−4^	0.3
Stone slag mixture	2100	2250	580	0.28	0.85	0	41.5	250	0.25	1700	1.0 × 10^−3^	0.5
Overburden	2200	2300	1050	0.39	0.83	30	49.1	100	0.25	2415	8.23 × 10^−3^	0.51

*ρ* is the density (kg/m^³^); *ρ*_*sat*_ is the saturated density (kg/m^³^); *K* is the modulus number; *n* is the modulus exponent; *R*_*f*_ is the failure ratio; *c* is the cohesion (kPa); *φ* is the friction angle (°); *K*_*b*_ is the bulk modulus number; *m* is the bulk modulus exponent; *K*_*ur*_ is the unloading–reloading modulus number; *k* is the hydraulic conductivity (m/s); and *J* is the allowable hydraulic gradient.

**Table 2:** Linear elastic material parameters.

Parameter	*ρ*(kg/m^3^)	*ρ*_*sat*_	*E* (GPa)	*μ*	*c*	*φ*	*k (m/s)*	*J*
geomembrane	2000	/	0.10	0.25	200	28.7	1.0 × 10^-12^	60.0
Concrete impermeable wall	2450	/	22.5	0.167	200	28.7	1.0 × 10^−8^	50.0
Micro-new bedrock	2500	2750	20.0	0.2	1000	45	1.0 × 10^−7^	4.0
Weathered layer	2600	2700	17.9	0.26	3600	51.58	1.0 × 10^−6^	2.9

*ρ* is the density (kg/m^³^); *ρ*_*sat*_ is the saturated density (kg/m^³^); *E* is Young’s modulus (GPa); *μ* is Poisson’s ratio; *c* is the cohesion (kPa); *φ* is the friction angle (°); *k* is the hydraulic conductivity (m/s); and *J* is the allowable hydraulic gradient.

Because the lithology is relatively uniform, hydraulic conductivity is assumed to be isotropic within each foundation stratum and cofferdam zone. Values for the foundation units were selected from the hydrogeological investigation, whereas those for the fill materials were established by reference to comparable projects. The adopted permeability coefficients are included in Tables 1 and 2. For the soil-wall contact model, a friction coefficient of 0.19 is used on the basis of engineering experience and the available interface studies.

### 4.3 Computational scheme

To ensure a consistent comparison, all other calculation conditions, including the foundation geometry, material parameters, boundary conditions, initial water levels, cutoff-wall penetration ratios, and drawdown process, were kept identical for the two schemes; only the arrangement of the seepage-control system was changed. The numerical analysis was conducted in three stages. First, steady coupled seepage–stress analyses were performed for both the core-wall and inclined-wall schemes with the cutoff wall penetrating 50%, 70%, and 100% of the overburden thickness. Second, transient coupled analyses were conducted during upstream water-level drawdown from 3,057.90 m to 3,036.90 m. During the coupled solution, pore pressure affected the effective stress and deformation, while the resulting volumetric strain updated the porosity and permeability for the subsequent seepage calculation. Third, the resulting stress and pore-pressure fields were used to evaluate the cutoff-wall response and slope stability under the selected condition. The slope stability was evaluated using the strength reduction method. In this method, the shear strength parameters of the soil are progressively reduced according to $c_{r}=c/F_{s}$ and $\text{tan}\emptyset_{r}=\text{tan}\emptyset /F_{s}$, where c and ∅ are the original cohesion and friction angle, $c_{r}$ and $\emptyset_{r}$ are the reduced strength parameters, and $F_{s}$ is the strength reduction factor. The reduction factor is gradually increased until the slope reaches the limit state, and the corresponding value of $F_{s}$ is taken as the factor of safety. The resulting pore pressures and stresses were then used in the slope-stability evaluation for the rapid-drawdown condition.

## 5 Analysis of calculation results

### 5.1 Analysis of seepage calculation results

Fig 8 presents the total-head fields obtained for the three cutoff-wall depths. In both structural layouts, equipotential lines crowd within the geomembrane and the foundation cutoff wall, confirming that these components carry most of the hydraulic drop. Increasing wall penetration progressively depresses the downstream phreatic surface. For the core-wall scheme, the head losses across the composite barrier are about 24.9, 27.9, and 36.9 m for penetration ratios of 50%, 70%, and 100%, respectively; the corresponding values for the inclined-wall scheme are 21.9, 25.9, and 30.9 m. Full penetration also lowers the seepage exit point and eliminates the short bypass path beneath a suspended wall. It therefore provides the most effective hydraulic connection between the geomembrane and the underlying low-permeability foundation.

**Fig 8:**
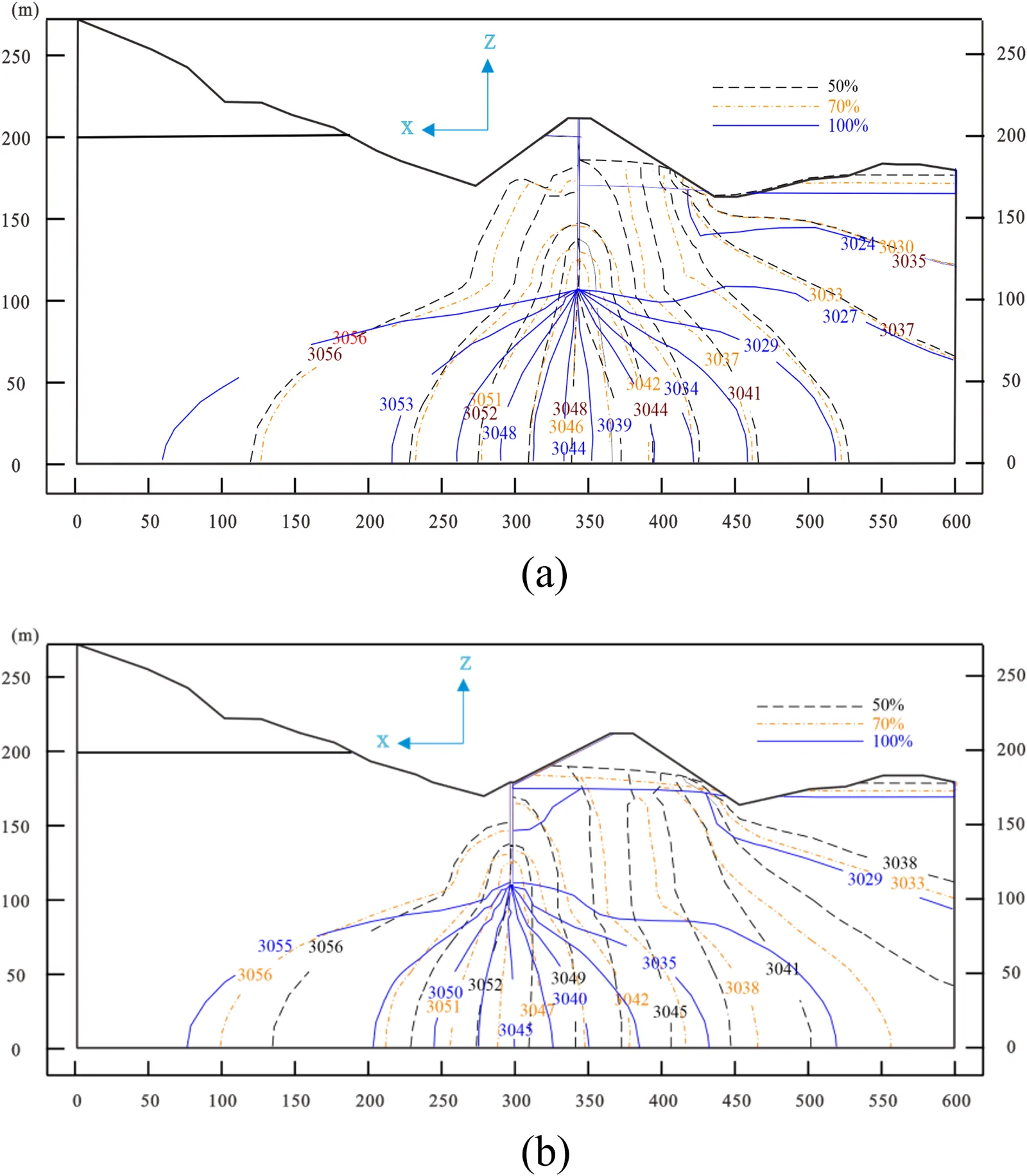
Comparison of total water head isolines of two types of earth-rock dam cofferdams: (a) Core wall scheme; (b) Inclined wall scheme.

The discharge comparison in Fig 9 shows a monotonic reduction in seepage as the cutoff wall is extended. For the core-wall arrangement, the calculated total discharge equals 0.068%, 0.042%, and 0.011% of the long-term mean river flow for 50%, 70%, and full penetration, respectively. The inclined-wall arrangement gives slightly larger ratios of 0.073%, 0.047%, and 0.015%. Thus, although both schemes provide substantial seepage control, the core-wall scheme consistently transmits less water for the same cutoff-wall depth.

**Fig 9:**
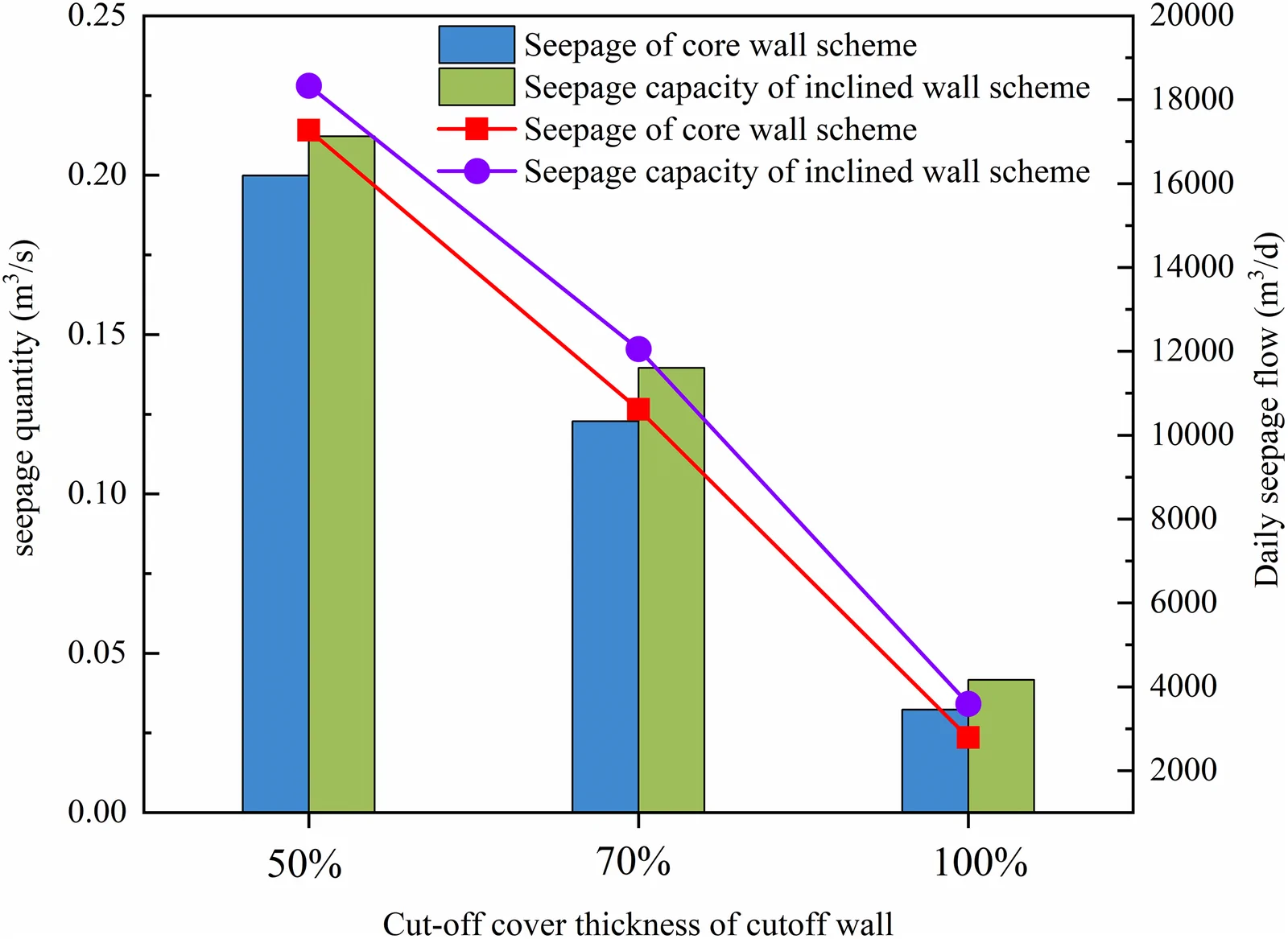
Comparison of seepage rate.

Hydraulic gradients at the principal material zones are compared in Fig 10. In Fig 10, A denotes the downstream seepage-exit zone; B and C denote the upstream and downstream foundation zones adjacent to the cutoff wall, respectively; and D and E denote the upstream and downstream sides near the cutoff-wall toe, respectively. The largest values occur in and around the cutoff wall, particularly near its toe, at the wall-rock contacts on the two abutments, and close to the downstream seepage outlet. This distribution reflects flow concentration as water is forced to detour around the impermeable barrier. All calculated gradients remain below the allowable values of the corresponding materials. Relative to the inclined-wall scheme, the core-wall scheme produces 28.92%, 22.50%, and 28.89% less seepage for the three penetration depths and also achieves a greater downstream head reduction. On hydraulic performance alone, the composite geomembrane core wall is therefore the more efficient arrangement.

**Fig 10:**
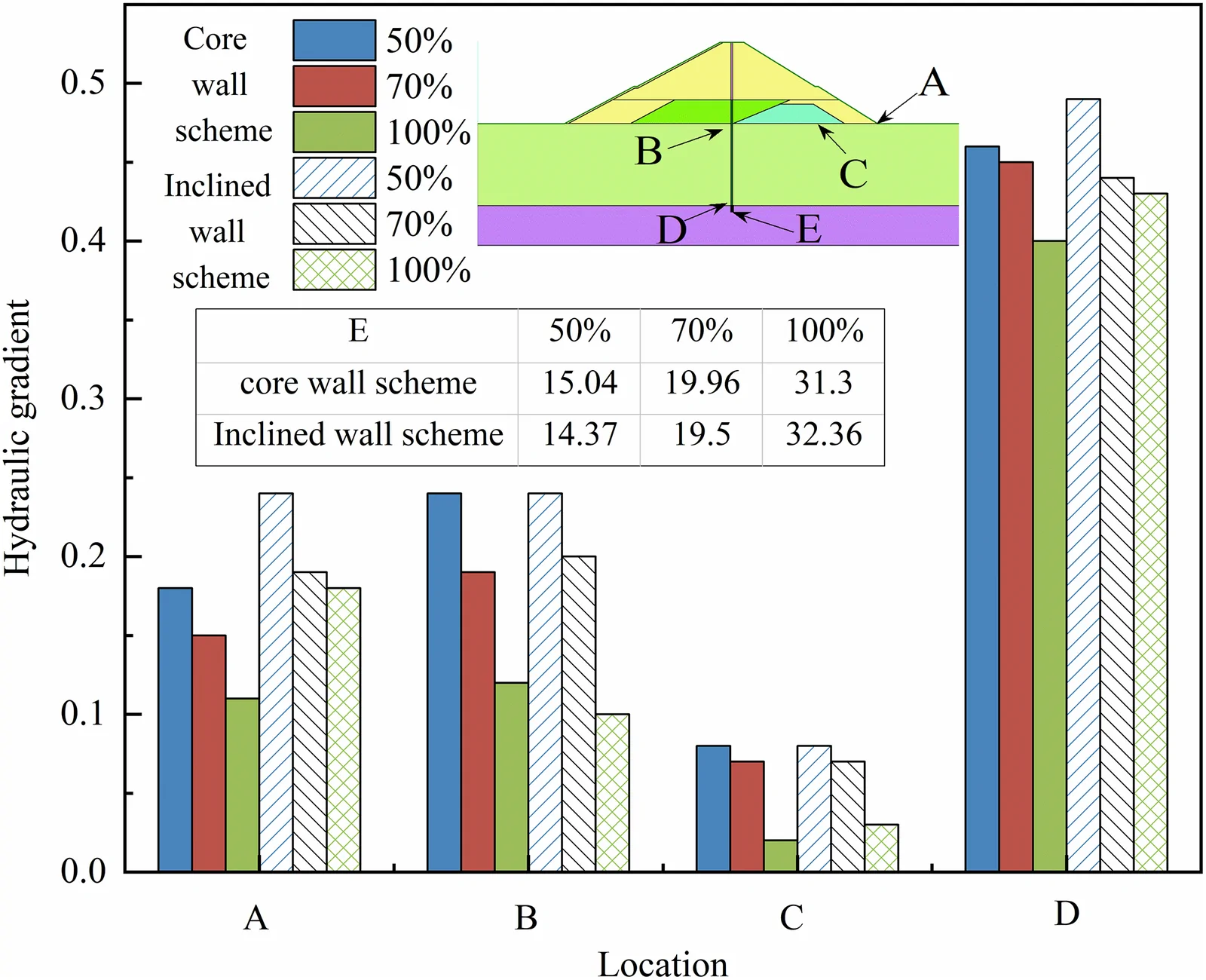
Comparison of hydraulic gradients.

Transient results for a drawdown rate of 5 m/day are plotted in Fig 11. As the upstream level falls from 3,057.9 m to 3,036.9 m, the phreatic surface inside the upstream shell does not follow the reservoir instantaneously; instead, it remains elevated and develops a convex profile. Continued drainage gradually dissipates the retained pore water and moves the phreatic surface downward, while the downstream shell remains close to the downstream boundary level. The peak hydraulic gradient occurs when the reservoir reaches 3,036.9 m and equals 35.05 for the core-wall scheme and 36.77 for the inclined-wall scheme. Thereafter, gradients in both the fill and the cutoff wall decline toward a stable condition.

**Fig 11:**
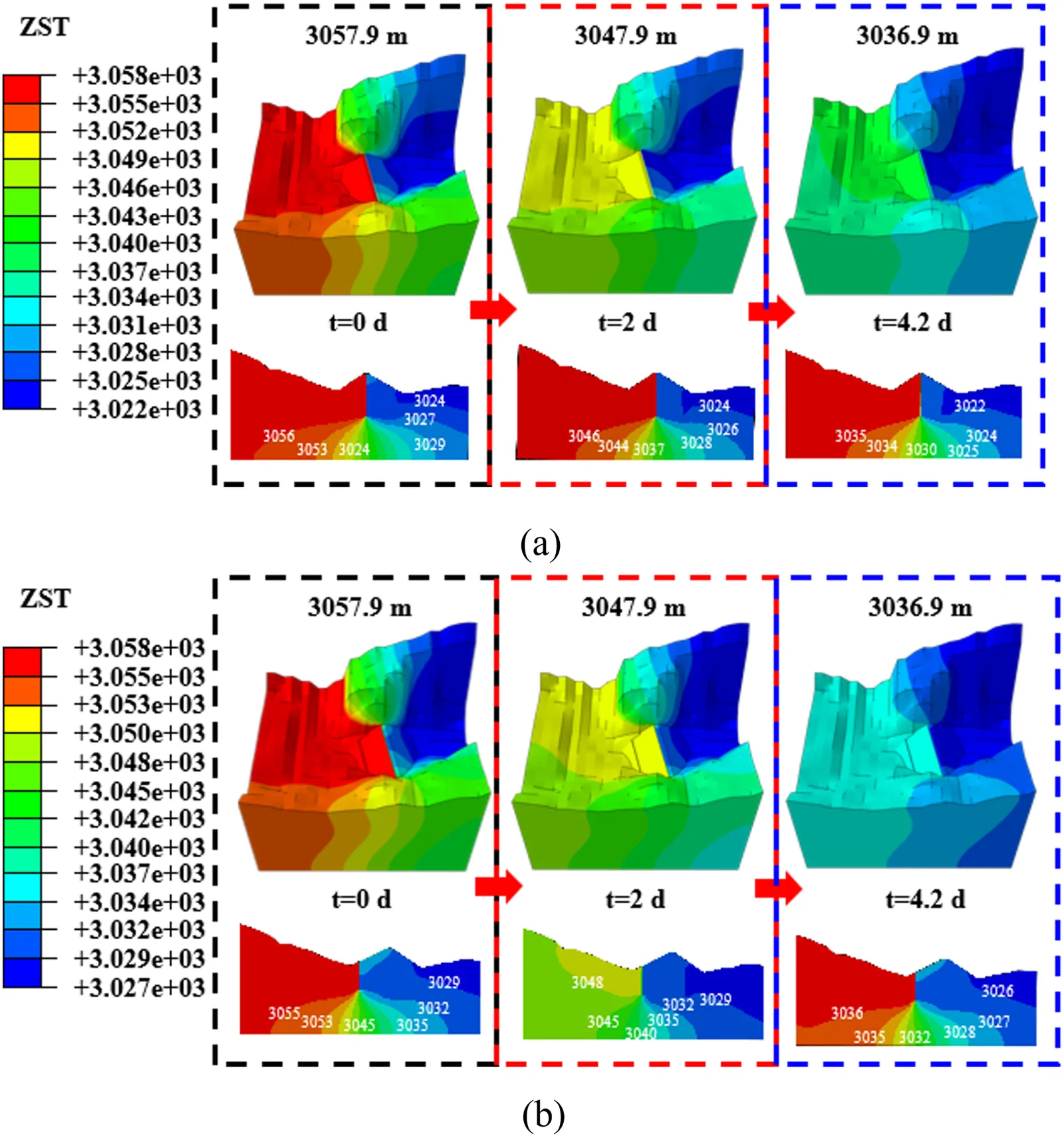
Isogram of total head of unsteady seepage field (Unit: m): (a) Core wall scheme; (b) Inclined wall scheme.

The difference in seepage-control performance between the two schemes is mainly related to the spatial arrangement of the geomembrane–cutoff-wall system. In the core-wall scheme, the geomembrane and cutoff wall form a more continuous central barrier, which concentrates the hydraulic head loss and lengthens the effective seepage path through the foundation. In contrast, the inclined-wall scheme redistributes the seepage path toward the upstream side, resulting in a relatively weaker overall reduction in seepage under the same cutoff-wall penetration depth.

### 5.2 Stress analysis

Using the seepage solution and the selected cutoff-wall depth, three-dimensional stress-deformation analyses were conducted for both structural layouts. Calculations with and without seepage loading were compared to isolate the hydraulic contribution. As shown in Fig 12, the two cofferdams develop similar stress patterns: the fill remains predominantly in compression, and stress increases in a layered manner toward the foundation, with the largest values in the lower stone-slag zone. No meaningful tensile zone forms within the cofferdam body. The core-wall scheme carries the higher overall stress level, with the corresponding compressive stress magnitudes reaching 1.15 MPa and 0.49 MPa, respectively.

**Fig 12:**
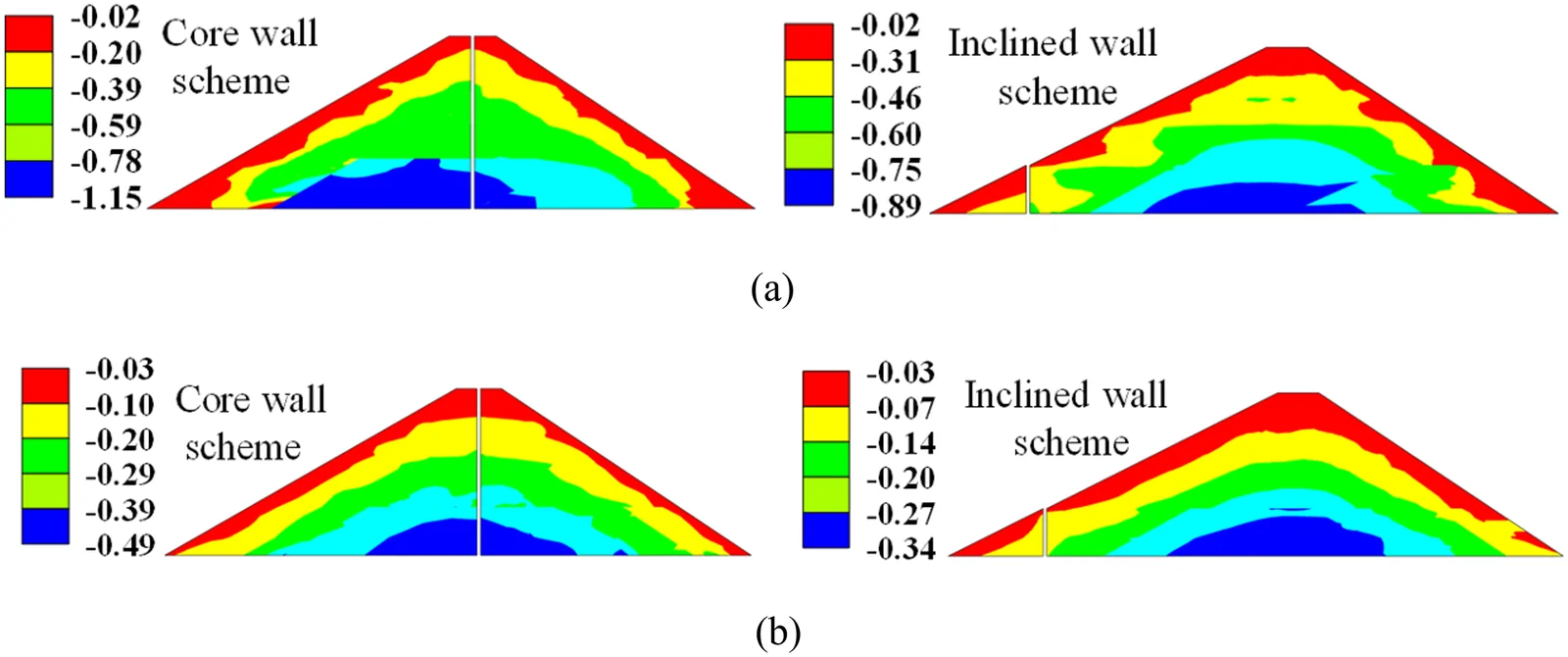
Isogram of stress distribution of cofferdam considering seepage (MPa): (a) Maximum principal stress; (b) Minimum principal stress.

The cutoff-wall contours in Fig 13 also exhibit broadly similar distributions for the two schemes. Most of the wall is compressed, whereas localized tension appears near the two abutments and at portions of the wall-rock interface. Upstream water pressure bends the wall downstream after impoundment. The embedded part is strongly restrained by the bedrock, while the upper central portion has greater freedom to deform under the three-dimensional valley geometry. This incompatibility concentrates tension near the constrained ends. The maximum tensile stress is 1.05 MPa in the core-wall scheme and 0.91 MPa in the inclined-wall scheme. For both schemes, the peak tensile stresses are concentrated near the two abutments of the cutoff wall, where deformation is strongly restrained by the surrounding foundation. Both values are lower than the design allowable tensile stress of 1.5 MPa, indicating that the tensile stress of the cutoff wall remains within the allowable range under the analyzed conditions.

**Fig 13:**
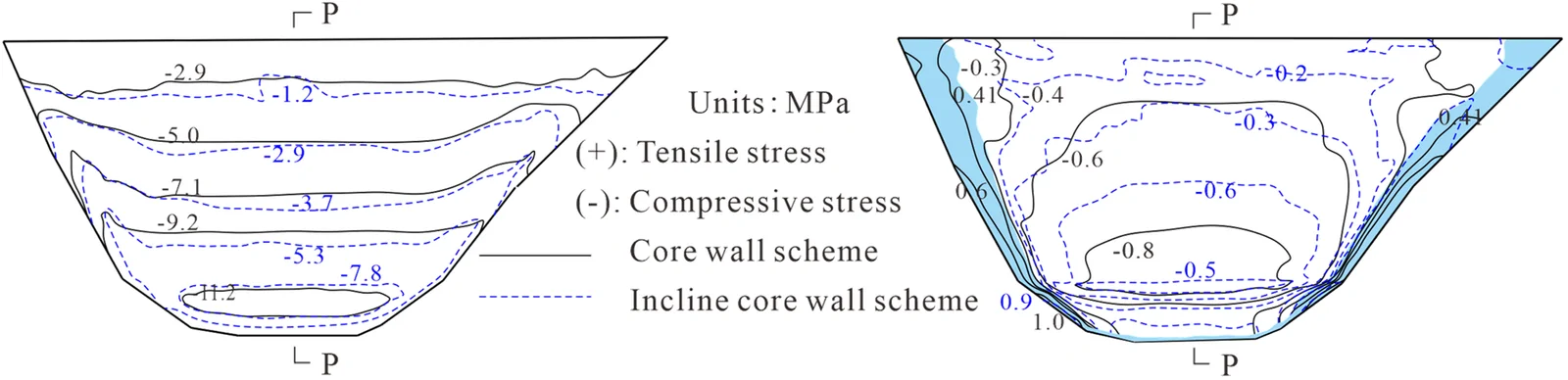
Isogram of stress distribution of cutoff wall considering seepage.

Fig 14 demonstrates that including seepage raises the stress demand on the cutoff wall, and that the extreme values remain larger for the core-wall scheme. The compressive stress does not increase continuously to the wall base. Instead, its magnitude increases with depth, reaches approximately 10 MPa near the neutral point, and then decreases toward the bottom. Above that point, wall self-weight and downward interface friction act in the same direction; below it, upward friction partially offsets the vertical load. The change in friction direction therefore governs the characteristic stress profile and explains why the seepage-coupled solution differs from the purely mechanical case.

**Fig 14:**
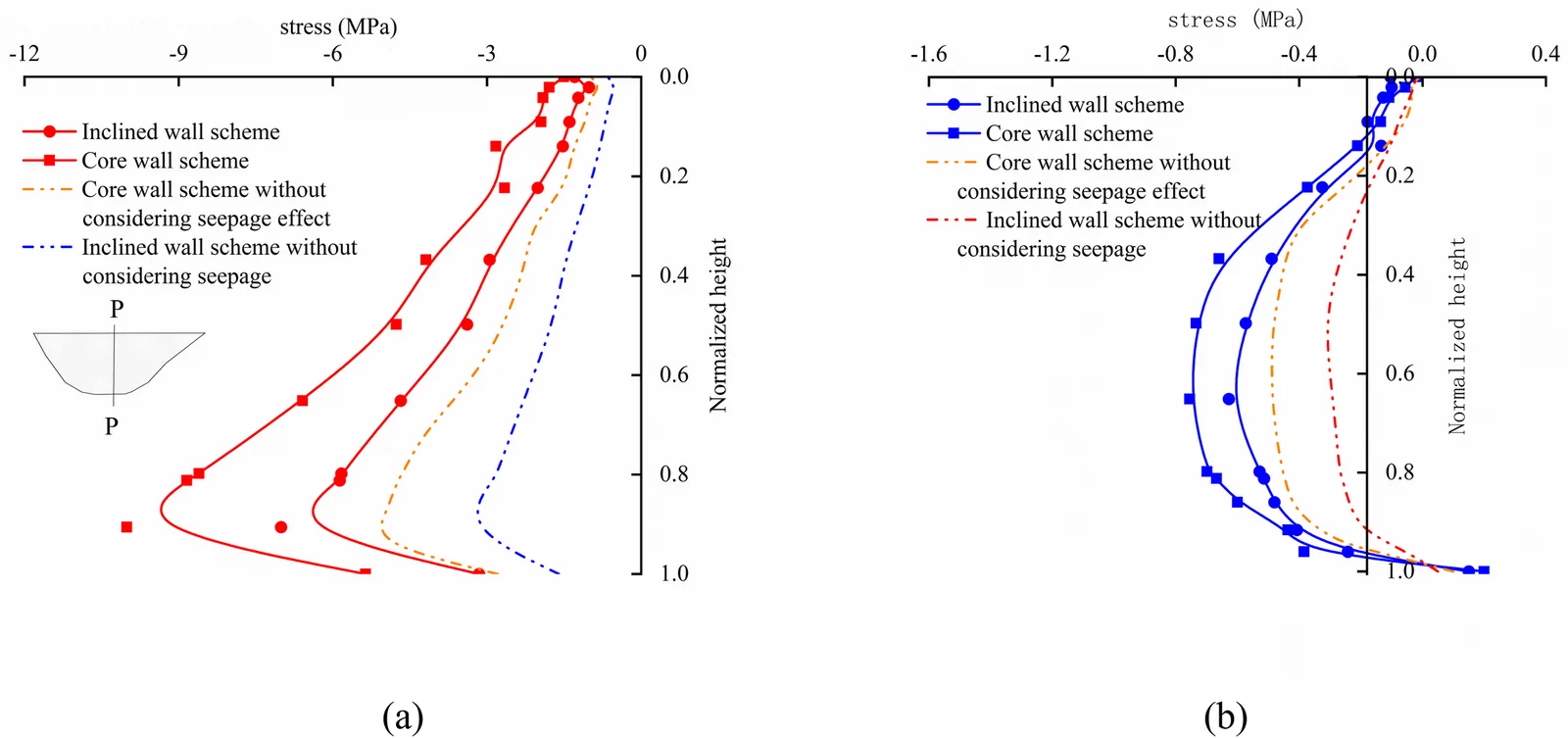
Influence of seepage on stress distribution of cutoff wall: (a) Maximum principal stress; (b) Minimum principal stress.

### 5.3 Stability analysis

Fig 15 shows the variation in the factors of safety of the riverbed section during drawdown from 3,057.90 m to 3,036.90 m. In the core-wall scheme, the upstream-slope factor decreases from 1.54 to a minimum of 1.446 and subsequently recovers to about 1.46; the downstream value remains close to 1.492. The initial reduction is caused by the mismatch between the rapidly falling external water level and the slower dissipation of pore pressure within the upstream shell. Stabilizing hydrostatic support is removed while an adverse seepage force persists, thereby increasing the driving force for sliding. As drainage proceeds, the reverse seepage weakens and the safety factor partially recovers. For the inclined-wall scheme, the upstream factor falls from 3.55 to 1.70, while the downstream value is approximately 1.489. Although rapid drawdown reduces stability in both cases, the inclined-wall scheme retains the larger upstream safety margin throughout the critical period.

**Fig 15:**
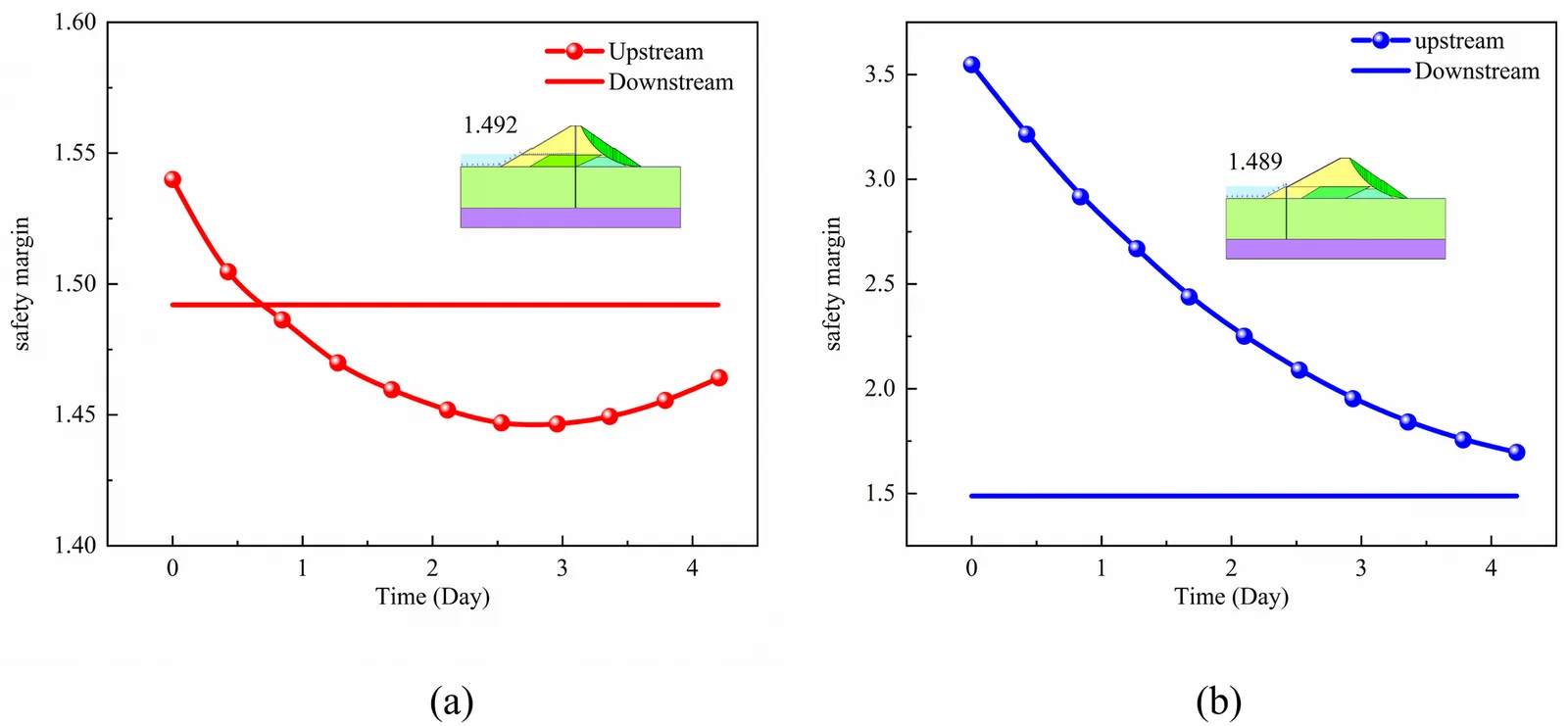
Variation in slope factors of safety during rapid drawdown: (a) Core wall scheme; (b) Inclined wall scheme.

## 6 Conclusions

A coupled seepage-stress analysis was used to compare composite geomembrane core-wall and inclined-wall schemes for an earth-rock cofferdam founded on deep overburden. The study considered cutoff-wall penetration, transient drawdown, structural stress response, and slope stability. The principal findings are summarized below.

(1) Extending the cutoff wall through the full overburden thickness most effectively interrupts the foundation seepage path, lowers the downstream head and phreatic surface, and reduces discharge to a very small fraction of the natural river flow. At every penetration ratio considered, the core-wall scheme yields less seepage than the inclined-wall scheme, indicating that the layout of the seepage-control scheme has an important influence on the overall seepage-control performance.
(2) The two schemes produce comparable overall stress patterns in the cofferdam and cutoff wall, with compression dominant in both structures. Local tensile zones occur at the cutoff-wall abutments and wall-rock contacts because of bending and deformation restraint. The core-wall scheme develops slightly higher tensile stress and higher overall cutoff-wall stress than the inclined-wall scheme, particularly when seepage effects are included.
(3) Rapid drawdown has little influence on the downstream-slope safety factor but temporarily reduces the stability of the upstream slope because pore pressure dissipates more slowly than the external water level falls. The core-wall scheme shows an initial decrease followed by partial recovery, whereas the inclined-wall scheme maintains a higher upstream safety factor over the analyzed drawdown period. These results indicate a trade-off between seepage-control performance and rapid-drawdown stability when selecting the seepage-control scheme.

This study is based on a specific cofferdam project and deterministic material parameters, and the conclusions may therefore be influenced by site-specific geological and hydraulic conditions. Future studies should further consider the spatial variability of foundation properties, long-term seepage evolution, and a wider range of construction and drawdown conditions to evaluate the applicability of the present findings.
